# VOICES OF LOW-INCOME LATINO OLDER ADULTS: WHAT ARE THEIR PERCEIVED LEARNING IMPEDIMENTS?

**DOI:** 10.1093/geroni/igad104.3692

**Published:** 2023-12-21

**Authors:** Tania Rodriguez, Rachel Wu

**Affiliations:** University of California Riverside, Riverside, California, United States; University of California Riverside, Riverside, California, United States

## Abstract

Cognitive and functional health disparities between Latino and White older adults show that Latinos have a higher risk for developing Alzheimer’s and related dementias (ADRD) in the United States. Such risk compromises Latino’s likelihood of becoming healthy agers, which is of high concern as they are one of the largest and fastest-growing minoritized groups in the United States. Nonetheless, there is a lack of research aiming to understand how to increase their cognitive and functional abilities. One way of stimulating cognitive and functional growth is through learning of new skills. However, learning is a privilege, and this population may face unique barriers to learning that other racial/ethnic groups may not, especially if they have a low income. This qualitative study focused on identifying the barriers to novel skill learning engagement of low-income Latino older adults. A total of 20 low-income, Latino older adults were interviewed in Spanish over the phone utilizing a semi-structured interview protocol. Themes were derived from the data using a thematic analysis approach. Our results showed that the following barriers were most common among our participants: health problems (80%), lack of money (75%), lack of awareness/knowledge of learning opportunities (75%), lack of technology access/literacy (75%), lack of proficiency in the English language (65%), lack of reliable transportation (55%), and lack of motivation due to being “old” (50%). These findings help inform learning program leaders, policymakers, and future learning interventions on the barriers that should be addressed for minoritized populations.

